# Replica Symmetry Breaking in a Weakly Scattering Optofluidic Random Laser

**DOI:** 10.1038/s41598-020-59575-2

**Published:** 2020-02-14

**Authors:** Anirban Sarkar, B. N. Shivakiran Bhaktha, Jonathan Andreasen

**Affiliations:** 1 Department of Physics, Indian Institute of Technology Kharagpur, Kharagpur, 721302 India; 2 Université Grenoble Alpes, Laboratoire Interdisciplinaire de Physique, F-38000 Grenoble, France; 3 Georgia Tech Research Institute, Atlanta, Georgia 30332 USA

**Keywords:** Lasers, LEDs and light sources, Optofluidics

## Abstract

We report the observation of replica symmetry breaking (RSB) in a weakly scattering optofluidic random laser (ORL). Coherent random lasing is indicated by the presence of narrow peaks rising out of the spectral background. This coherence helps to identify a random laser threshold, which is expected to be gradual with weak scattering. We find that lasing action initiated using optical pulsed pumping coincides with the onset of both RSB and Lévy flight statistics. However, the transition from the photonic paramagnetic to photonic glass phase is more subtle in that the Parisi overlap function broadens instead of completely changing shape. This subtlety is balanced by an accompanying result of identical experimental conditions giving rise to lasing or no lasing depending on the shot. Additional statistical calculations and investigations into the fundamental physical mechanisms present in the ORL support this conclusion. Using simple numerical models, we study the critical spectral properties required for RSB to occur, as indicated by the Parisi overlap function. The simplicity of the models helps demonstrate the sensitive nature of this tool and the necessity of additional verification of the physical mechanisms present in the experiment.

## Introduction

Random lasers are a special class of active disordered materials where laser oscillations survive due to the optical feedback provided by multiple scattering^1,2^. Random lasers are spectrally distinguished from conventional lasing systems by randomly distributed sharp spikes over the emission. These sharp spikes can be directly attributed to resonances known as quasi bound (QB) states (e.g.^3,4^) of the passive cavity when the strength of scattering is strong^5^. On the contrary, weakly scattering systems exhibit spatially extended QB states. One-to-one correspondence exists between QB states and lasing modes only at the lasing threshold, only if that threshold is small enough^5^, and only if the pumped region includes the entire spatial area over which the QB states extend^6^. When such correspondence breaks down, lasing modes can be described as a mixture of QB states; for example, as scattering becomes very weak. Despite the absence of correspondence with a single QB state, these lasing modes retain the spatial properties of the QB states from which they are composed. Such states exhibit strong spatial overlap, short lifetimes, and strong leakage at the boundary due to their extended nature. This gives rise to mode competition and strong inter-modal coupling via spatial hole burning^7,8^, which can result in chaotic behavior with different emission spectra observed pump shot to pump shot^9^.

Insight into shot-to-shot fluctuations has been provided by spin glass theory^10,11^. The replica symmetry breaking (RSB) phase transition was observed experimentally^12^ and the theoretical connection between intensity and complex amplitude fluctuations established^13^. According to spin glass theory, the statistical distribution of an overlap parameter *q*, namely the Parisi overlap function *P*(*q*), changes its shape in the presence of a large number of competing equilibrium states. This means that identical copies of the system realized under identical experimental conditions reach different sets of equilibrium configurations. In disordered systems, random laser modes are treated as the spin variable and the excitation energy plays the role of inverse temperature. When the pump intensity is low (i.e., temperature is high), the system is either below the lasing threshold, in the single mode lasing regime, or multiple non-interacting (spatially separated) modes are excited independently. As the pump intensity increases (i.e., temperature reduces), the modes compete with one another to survive due to spatial hole burning and, in the RSB regime, lasing modes oscillate in a correlated manner. In random lasers, disorder frustrates the system from achieving a lower energy state of degenerate modes.

A strong connection between the spin-glass phase and Lévy flight statistics was reported recently^14^. Intentionally avoiding static realizations of random scatterers, RSB was later observed without Lévy statistics^15^. It is unclear exactly how replica symmetry can be broken without persistent identical systems, however, any common mechanisms and the precise interdependence of these effects does remain elusive^16^.

**Figure 1:**
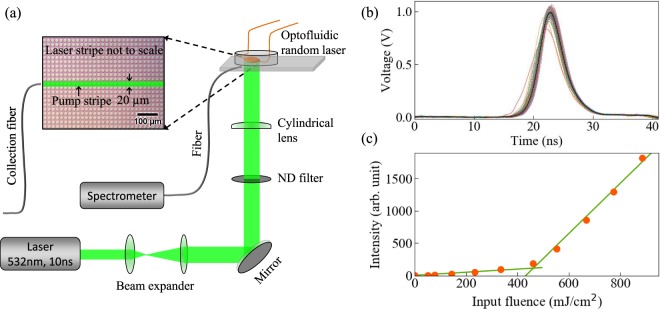
The schematic of the experimental setup with replicas of the excitation pulse and the laser threshold. (**a**) The ORL is pumped by a ns laser with a stripe pump geometry. The random laser emission is recorded using a fiber coupled spectrometer. The inset shows the optical microscope image of a portion of the dye-filled ORL, where the white dots are the scatterers of random sizes. (**b**) Temporal profiles of the pump pulse (100 shots) used to excite the ORL are shown. The pump profiles are nearly identical due to small fluctuations. Standard deviations of 1.04% confirm the replica nature of the pump, which is a necessary condition to replicate the experiment. (**c**) ORL emission vs input fluence is plotted, which demonstrates the typical laser threshold behavior. ORL threshold is obtained at *P*_*th*_ = 433.04 mJ/cm^2^.

Here, we study the weakly scattering optofluidic random laser (ORL), shown in Fig. 1(a). In optofluidics, optical properties of fluids are studied by infiltrating them in microfluidic channels^17,18^. Lab-on-a chip integration, mechanical stability, straightforward manipulation of fluid, and inexpensive mass production facilities make them attractive systems for future use. These ORLs avoid any issues raised by non-identical realizations of randomness, since the scatterers are fixed. Inherent randomness, present in the structures due to limitations of the fabrication process, is exploited as a fixed realization of disorder over many pump pulses. Typical limitations of laser dye, such as bleaching, are overcome by constant replenishment due to a continuous flow^19^. However, the dye flow does not make the scatterers dynamic and the gain is well described by a static effective susceptibility. Further details of this device and its potential applications have been published elsewhere^20^.

Because direct measurement of random laser mode time evolution is not yet possible, the indirect usage of emission intensity spectra to demonstrate RSB is appealing in its ease of use. Using this method, RSB has been observed in systems without disorder but with interacting lasing modes^21,22^. As noted elsewhere^23,24^, mode interaction as opposed to randomness alone may be the major factor that controls RSB. However, modes may interact without phase correlations, which is an added requirement of RSB^13^, and for which the Parisi overlap function *P*(*q*) alone offers experimental verification, thus far. The indirectness of *P*(*q*) implies that other physical mechanisms, which might cause similar spectral fluctuations, may yield “false positives” for RSB. Therefore, we explore numerous spectral measurements in this paper as well as a few numerical models in order to verify the operational regime of the ORL and support our conclusion that RSB occurs.

We begin with an overview of the experimental setup and verification that replica conditions are satisfied. Coherent random lasing is then demonstrated and typical emission spectra presented. Spectral fluctuations and correlations between series of random laser emission pulses as well as Lévy flight statistics show clear gain saturation and suggest strong mode competition. The Parisi overlap function *P*(*q*) is calculated for pump fluences of interest and results compared against explicit mode correlations. We end by using a few simple numerical models to explore what impact various emission spectra qualities have on the Parisi overlap function, underscoring the necessity of verifying which physical mechanisms can and cannot exist in the experiment. Finally, we present our discussion of this spectroscopic study.

## Experiments and Results

The ORL was fabricated with polydimethylsiloxane (PDMS) following the soft lithography protocol^25^. Details of the fabrication process are described in the “ Methods” section. The device contained a circular reservoir of diameter $D=3$ mm with scatterers of diameters $d=17$ *μ*m distributed in a 2-D square array pattern with $\Lambda =20$ *μ*m periodicity. Standard deviation of $\sigma =433.1$ nm in the size of the scatterers, comparable to the wavelength of the light, appeared due to the fabrication limitation of the photo-lithography process. A solution of Rhodamine-6G dye in methanol at 2.5 mM concentration was used to provide gain.

Pumping was performed with a Nd:YAG nanosecond laser having a pulse width of ∼10 ns and emission wavelength of $\lambda =532$ nm. Temporal profiles of the pump pulse over 100 shots were recorded using a fast photodiode and are shown in Fig. 1(b). The standard deviation in the temporal profile of the pump was evaluated as 1.04%, which we find a reasonable replica nature of the pump. Global pumping here indicates the use of a single pump beam where gain exists over the entire spatial region of the pump spot. Outside the pump spot, light is absorbed by the laser dye, inducing confinement^26^. This effectively limits the spatial extent of the ORL to the pumped region plus the absorption length in the plane of the device.

A single line of scatterers in the ORL is pumped with a stripe measuring $L=1000$ *μ*m long and $W=20$ *μ*m wide, which was obtained using a cylindrical lens of $f=5$ cm focal length. Pump intensity was varied from a low value to a value above the lasing threshold of the system. In each case, a set of ${N}_{s}=100$ single shot emission spectra were collected using an optical fiber coupled to an Avantes spectrometer having a spectral resolution of 0.04 nm. A 100 ms time gap is maintained between the collection of two successive spectra to avoid the memory from the previous pulse excitation. The collection fiber was placed along the direction of the on-average periodic square lattice array of the ORL. For a particular pump intensity, since the position of the scatterers is fixed in the ORL, the system is identical under each laser pulse excitation.

We briefly note that the mean free path is estimated numerically^27^ as $\ell =8.6\pm 1.2$ mm over the wavelength range 500 nm $\le \lambda \le 600$ nm. All 1-D pump lengths *L* satisfy $L < \ell$, meaning the system is weakly scattering and approximately in the 1-D ballistic regime. One relevant consequence of weak scattering is that the lasing transition is expected to be more gradual than when stronger scattering is present^28^.

**Figure 2:**
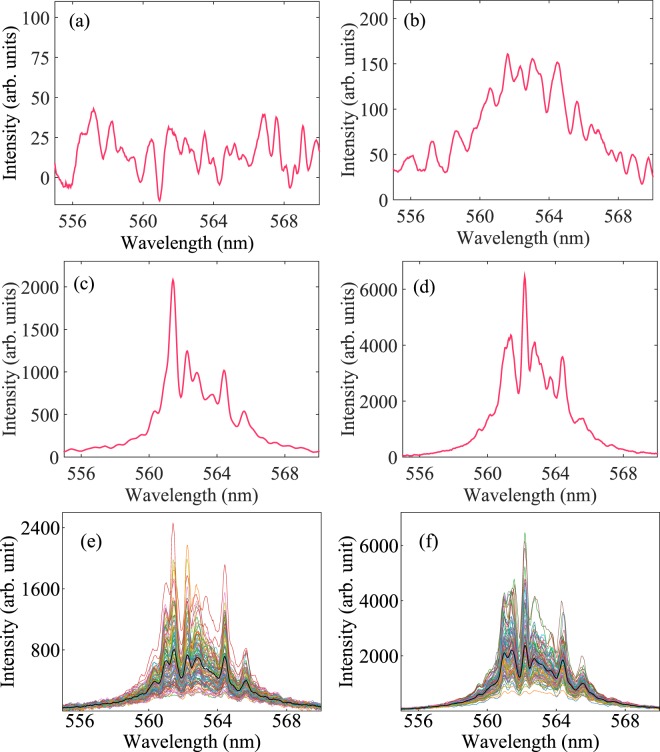
Emission spectra of ORL and their fluctuations. Typical single-shot ORL emission spectra recorded at four different pump fluences below ((**a**) 50.82 mJ/cm^2^, (**b**) 333.71 mJ/cm^2^) and above ((**c**) 552.94 mJ/cm^2^, (**d**) 774.16 mJ/cm^2^) the lasing threshold are shown. Below threshold, sharp spikes are absent in the emission spectra, while above threshold, they are randomly distributed. The origin of the narrow spikes is attributed to gain-altered resonances of the random medium. ${N}_{s}=100$ single-shot spectra of the ORL overlaid on average spectrum for pump fluences (**e**) 552.94 mJ/cm^2^, and (**f**) 774.16 mJ/cm^2^ are shown. Black curves represent the average intensities. Although intensities fluctuate from their average value, positions of the distinct lasing modes remain fixed.

On increasing the pump fluence, emission of the device exhibits typical lasing threshold behavior with an estimated threshold value ${P}_{th}=433.04$ mJ/cm^2^, as shown in Fig. 1(c). This threshold value is higher than those reported in other optofluidic random lasers^19,29,30^ and can be attributed to the partial pumping configuration discussed above, where long regions on either side of the pump stripe remain un-pumped. This result is consistent with reported studies on random lasers under partial pumping^6^. Moreover, our earlier report on a similar device^20^, with a different pumping configuration, demonstrates a lasing threshold comparable to the lower values expected. The emission spectrum is broad at low excitation energies Fig. 2(a) and becomes narrower close to the lasing threshold Fig. 2(b). In Fig. 2(a), the single shot emission spectrum has a poor signal to noise ratio. A broad spectrum with improved signal to noise ratio is obtained by averaging the single shot spectra acquired over a large number of shots. However, we present the single-shot spectra at all pump fluences to aid in the understanding of fluctuations and correlations that play a central role in RSB. Further increasing the pump fluence above threshold, randomly distributed sharp lasing peaks appear on top of a broad pedestal Fig. 2(c,d). These lasing peaks confirm the coherent emission properties expected from the device. More detailed investigations of random lasing properties of the ORL have been demonstrated earlier^20^.

## Fluctuations and Correlations

To investigate the fluctuations in the emission of the ORL, sets of ${N}_{s}=100$ single-shot spectra were recorded at each pump power. During acquisition, all the experimental conditions were kept constant to satisfy the replica condition. Two such sets of spectra along with their average have been shown in Fig. 2(e,f). In both the cases, strong fluctuations are observed around a discrete set of persistent modes. On averaging these fluctuating spectra, as shown, the spectrum retains signatures of these modes.

The fluctuations of the pedestal over which the modes appear (DC fluctuations) observed in Fig. 2(e,f) should be noted. This is a common observation in dye-based random lasers^31^ and qualitatively similar to those observed from incoherent random lasers^12,32^. Although sharp peaks indicating coherent modes appear consistently in the emission spectra, behavior similar to incoherent random lasers should not be surprising, since this scattering system is so weak (recall $\ell  \sim 8.6$ mm).

It is conceivable that such DC fluctuations stem from experimental inconsistencies. Therefore, we have verified to the best of our ability that the replica nature of the experiment was maintained. Variations in laser dye liquid flow rate, for example, might cause slightly different amplification rates from shot to shot. This could be caused by blockage or spatial inconsistencies in the scattering system, or other regions through which the laser dye flows. To check this, we monitored the movement of air bubbles and dust through the system prior to the ORL experiments and observed no pooling nor other discontinuous behavior. Another potential issue is that the dye can move at a slower rate compared to the rate at which it is being pumped (20 Hz). However, with continuous flow already observed, this would mean that the incoming flow rate of new dye molecules (to the pumped region) matches the outgoing flow rate. Therefore, the overall emission might be reduced compared to a case with only new dye molecules, but shot to shot conditions here should be identical.

**Figure 3:**
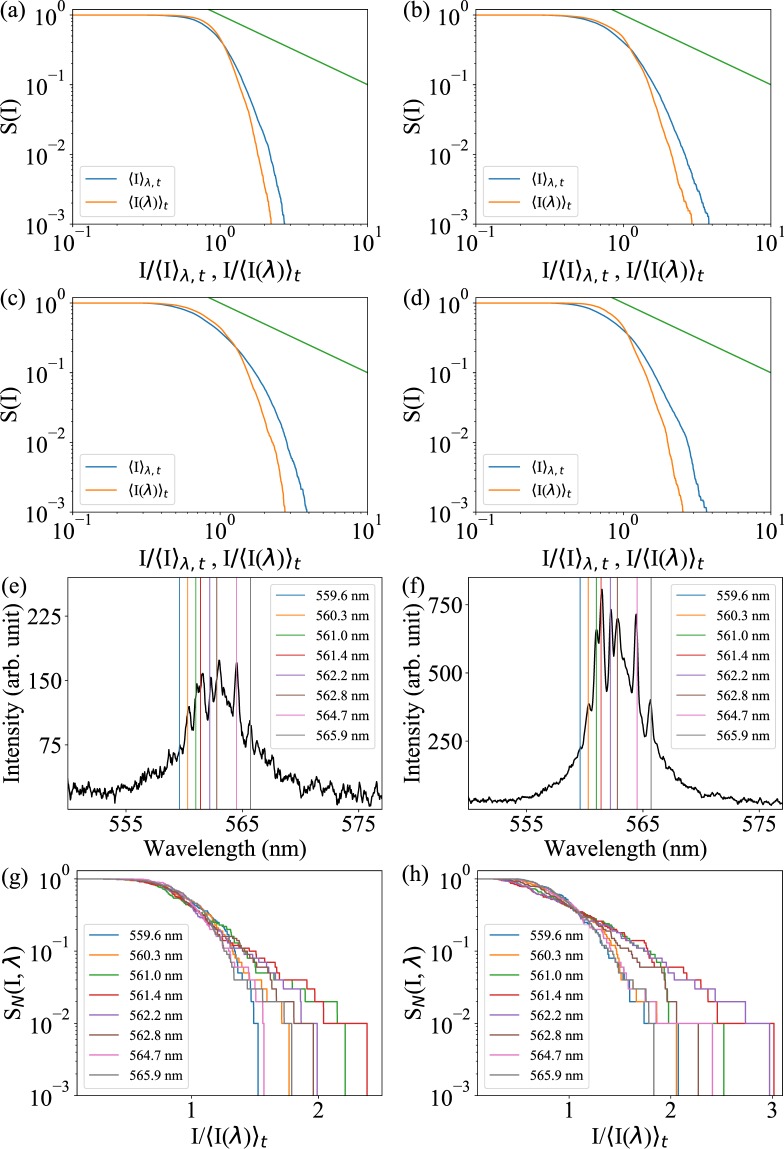
Survival function of the ORL emission. Survival function $S(I)$ pooled across wavelengths for pump fluences (**a**) 333.71 mJ/cm^2^, (**b**) 460.49 mJ/cm^2^, (**c**) 552.94 mJ/cm^2^, and (**d**) 774.16 mJ/cm^2^. Orange curve is obtained from the intensities pooled across wavelengths after normalizing by their corresponding shot-averaged value at each wavelength, while the blue curve is obtained from the intensities pooled across the wavelengths and shots before normalizing by their average value. A power-law line (green curve) with exponent −1 is shown for reference. (**e**,**f**) Sample emission spectrum and (**g**,**h**) lasing mode survival functions of the ORL at pump fluences: (**e**,**g**) 333.71 mJ/cm^2^ and (**f**,**h**) 552.94 mJ/cm^2^. Survival functions of the lasing modes show faster than the exponential decay due gain saturation in the ORL.

Next, intensity fluctuations of the overall emission spectra are quantified using the survival function *S*(*I*), which is calculated as the fraction of shots having emission intensity at a single wavelength higher than intensity *I*^33^. For the orange curves in Fig. 3(a–d), the intensity distribution is first normalized by the shot-averaged intensity at each wavelength and then pooled across the wavelengths. For the blue curves, the intensities are kept at their original values when combined together, then normalized by the shot- and wavelength-averaged intensity. The fact that these two curves are different at each pump fluence in Fig. 3(a–d) indicates the entire spectral intensity distribution contains non-trivial correlations.

The Lévy statistical regime is defined as one in which the probability distribution follows a power-law behavior. Such probability distributions also exhibit survival functions that follow a power law. If Lévy statistics are present in the ORL emission spectra, we expect a power-law decrease with an exponent of −1 in *S*(*I*) after it drops from 10^0^ until a high-intensity cutoff is reached. This behavior might exist for a very short range of normalized intensities in Fig. 3(a–d), particularly Fig. 3(c). But at best, the trend is unclear. We shall return to this issue later and employ a more sensitive method for clarity.

Looking at a persistent set of wavelengths with spectral peaks, the survival function ${S}_{N}(I,\lambda )$ is evaluated from the intensities normalized only by shot-averaged modal intensity and plotted on a log-linear scale in Fig. 3(e–h). In this case, an exponential decay of the survival functions would indicate Gaussian statistics^34^ and faster-than-exponential decay reveal gain saturation. Figure 3(e,g) show results just below the lasing threshold indicated by Fig. 1(c). The higher intensity modes near the center of the gain spectrum in Fig. 3(e) exhibits a more apparent exponential decay in Fig. 3(g) before decaying more quickly at higher normalized intensity values. This suggests these modes may already be lasing and experiencing gain saturation. Figure 3(f,h) show results just above the lasing threshold, where modes near the center of the gain spectrum more clearly demonstrate gain saturation and modes at the edges, particularly the mode at 559.6 nm, may not yet be lasing. Gain saturation is a critical physical mechanism that must be present for mode coupling to occur in the ORL.

Explicit correlations between lasing mode intensities (e.g., see^35,36^) are next quantified using the covariance *c*_2_, expressed at two different wavelengths ${\lambda }_{1}$ and ${\lambda }_{2}$ as

1 $${c}_{2}({\lambda }_{1},{\lambda }_{2})={\langle I({\lambda }_{1})I({\lambda }_{2})\rangle }_{t}-{\langle I({\lambda }_{1})\rangle }_{t}{\langle I({\lambda }_{2})\rangle }_{t}.$$

**Figure 4:**
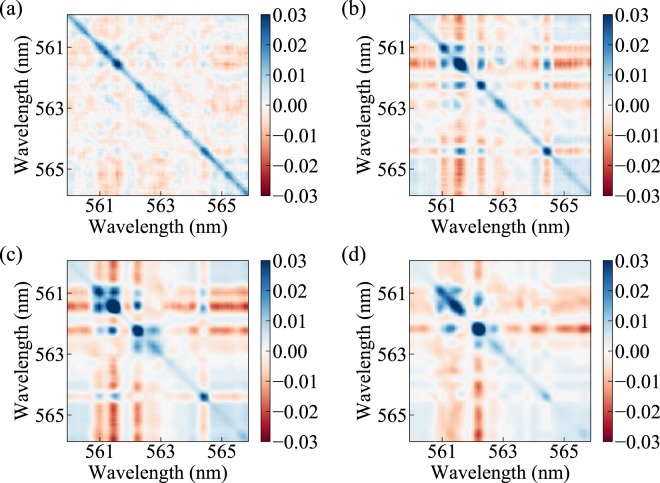
Cross-correlation map of the ORL modes. The correlation values are evaluated from the normalized spectral intensities at the pump fluences: (**a**) 333.71 mJ/cm^2^, (**b**) 460.49 mJ/cm^2^, (**c**) 552.94 mJ/cm^2^, and (**d**) 774.16 mJ/cm^2^. The diagonal elements represent the autocorrelations of individual modes, while the off-diagonal elements are the covariances across the modes.

Before calculating the covariance, the intensity of each spectrum is normalized by its average intensity. Figure 4 shows the covariance between modes at four different pump fluences near and above threshold. Diagonal positions in the covariance plots represent the auto-correlations of the modes. Near threshold in Fig. 4(a), the cross-correlations are much smaller than the auto-correlations. However, clear anti-correlations are observed in the red streaks separated by islands of blue, positive correlations. Thus, although likely to be present, mode competition appears weak at this pump fluence. Above the threshold pump power in Fig. 4(b–d), strong cross-correlations (both positive and negative) occur more clearly, demonstrating mode competition.

Toward additional verification that the physical mechanism for mode coupling is available, we first note that inhomogeneous broadening can enable simultaneous lasing in modes that spatially overlap, consesquently removing the possibility of mode competition. This is especially true in solid-state dye-doped polymer random lasers, where a relatively small homogeneous linewidth of 0.8 nm can be found^37^. In our system of Rhodamine-6G dye molecules in liquid methanol, the homogeneous linewidth is an order of magnitude larger, estimated at 8 nm with photon-echo spectroscopy^38^. This means that lasing modes spectrally separated by less than 8 nm *can* couple. The estimated width of the gain curve, provided by the relaxation and dephasing times *T*_1_ and *T*_2_, is 4.2 nm. Most of the ORL emission in Fig. 2 is observed in the range 559 nm to 567 nm. Therefore, with the physical mechanism for mode coupling present, results in this section provide evidence that the lasing modes compete with each other for gain.

### Lévy flight statistics

Because the evidence of Lévy statistics in the previous section is unclear, we examine the same ORL emission spectra again here using the exponent *α* calculated from the quantile-based McCulloch method^39,40^. This method is sensitive enough that it has been used as an identifier of the random lasing threshold^41,42^.

**Figure 5:**
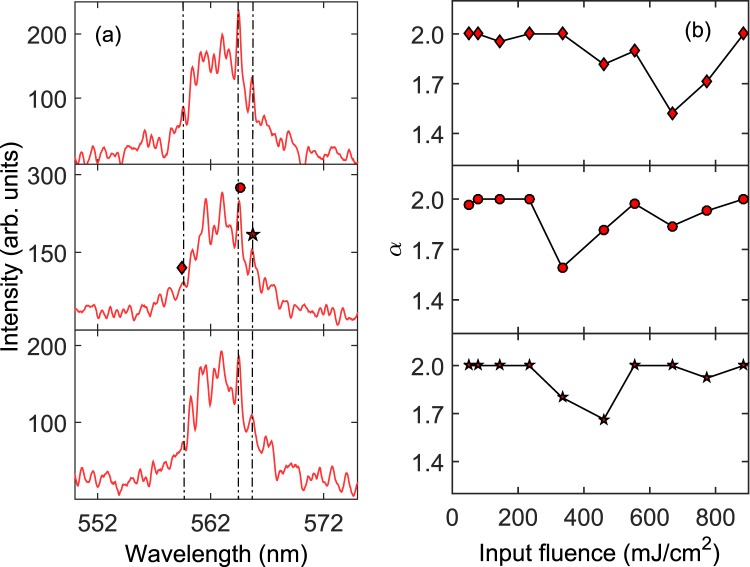
Lévy exponents of the lasing modes. (**a**) Three single-shot emission spectra shown at the pump fluence 333.71 mJ/cm^2^. Roughly 43 out of 100 single-shot spectra show these sharp spikes. Others look like the spectrum shown in Fig. 2(b). (**b**) Values of the Lévy exponent *α* calculated at the wavelengths, 559.6 nm, 564.7 nm, and 565.9 nm, indicated by vertical lines in (**a**).

Three wavelengths, where sharp peaks appear, were chosen to calculate *α* and are shown in Fig. 5(a). Clear transitions from the Gaussian ($\alpha =2$) to Lévy statistical regime ($0 < \alpha  < 2$) are observed at different pump fluences close to threshold in Fig. 5(b), depending on the mode. The Lévy regime is reached at the fluence 333.71 mJ/cm^2^ for the wavelengths 564.7 nm and 565.9 nm and later at fluence 460.49 mJ/cm^2^ for the wavelength 559.6 nm. Because the wavelength 559.6 nm is further from the gain center wavelength, it is expected to reach its lasing threshold at a higher pump fluence so that the delayed occurrence of Lévy statistics is not surprising. Based on recent comments concerning the coincidence of Lévy statistics and that of the glassy state of light^24,43^, we will compare the average value of the exponent $< \alpha  >$ over all lasing modes to the Parisi overlap function in the next section.

The pump fluence 333.71 mJ/cm^2^ is below the lasing threshold according to Fig. 1(c) but particular wavelengths reveal a signature of Lévy statistics, as demonstrated above. Although the single-shot spectrum in Fig. 2(b) is representative of this pump fluence, 43 of the 100 spectra show sharp peaks, three of which are shown in Fig. 5(a). While the typical definition of laser threshold at 433.04 mJ/cm^2^ shown in Fig. 1(c) remains true on average, more in-depth statistical calculations reveal another relevant lasing threshold is reached at 333.71 mJ/cm^2^. This is a somewhat extreme example of different emission characteristics rising from identical experimental conditions. Because mode coupling is weakly indicated by Fig. 4(a) and Lévy statistics are observed here at wavelengths close to the gain center wavelength, this begs the question if RSB is also occuring at the pump fluence 333.71 mJ/cm^2^.

### Parisi overlap function

The intensity correlation function, or overlap parameter, is defined as

2 $${q}_{\gamma \beta }=\frac{{\sum }_{{\lambda }_{k}=1}^{N}{\Delta }_{\gamma }({\lambda }_{k}){\Delta }_{\beta }({\lambda }_{k})}{\sqrt{{\sum }_{{\lambda }_{k}=1}^{N}{\Delta }_{\gamma }^{2}({\lambda }_{k})}\sqrt{{\sum }_{{\lambda }_{k}=1}^{N}{\Delta }_{\beta }^{2}({\lambda }_{k})}},$$

where $\gamma ,\beta =1,2,\mathrm{3...}.,{N}_{s}$ and *N* is the number of spectral points. The average emission intensity at a wavelength indexed by ${\lambda }_{k}$ is expressed as

3 $$\bar{I}({\lambda }_{k})=\mathop{\sum }\limits_{\gamma =1}^{{N}_{s}}{I}_{\gamma }({\lambda }_{k})/{N}_{s}$$

and the intensity fluctuations are evaluated by

4 $${\Delta }_{\gamma }({\lambda }_{k})={I}_{\gamma }({\lambda }_{k})-\bar{I}({\lambda }_{k}).$$

**Figure 6:**
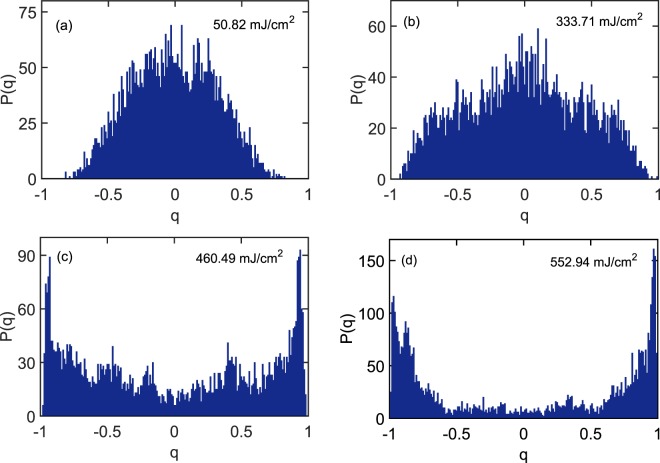
Distribution of the Parisi overlap parameter. For ${N}_{s}=100$ laser shots, the probability density functions of the overlap parameter in an ORL are shown at four different pump fluences: (**a**) 50.82 mJ/cm^2^, (**b**) 333.71 mJ/cm^2^, (**c**) 460.49 mJ/cm^2^, and (**d**) 552.94 mJ/cm^2^. Change in the shape of distribution function upon increasing the pump fluence is intended to indicate the spin-glass transition.

The statistical distribution of the overlap parameter is calculated as *P*(*q*) and, according to spin-glass theory, its shape describes the material phase observed. The value of *q* at which *P*(*q*) is maximized is defined as ${q}_{max}$. In the photonic paramagnetic phase ${q}_{max}=0$, while in the spin-glass phase ${q}_{max}\ne 0$. In Fig. 6, the ORL exhibits the photonic paramagnetic phase at low pump power and the spin-glass phase at higher pump power. From Fig. 6(a) it is clear that ${q}_{max}=0$, where the system is clearly in its fluorescence phase and no population inversion exists, meaning no random laser modes are excited. Interestingly, Fig. 6(b) shows that ${q}_{max}$ is still near zero for the pump fluence 333.71 mJ/cm^2^, which would indicate the ORL remains in the photonic paramagnetic phase. However, results by Antenucci *et al*. show that *P*(*q*) obtains a “non-trivial” distribution as the random laser threshold is crossed^13^. Their numerical model predicts the presence of smaller-amplitude side lobes near threshold, which we expect to effectively broaden *P*(*q*). Indeed, compared to Fig. 6(a), the distribution is broadened and shoulders appear on the distribution near $|q| \sim 0.5$. Together with the evidence of mode competition and Lévy statistics at this pump fluence, we conclude that the onset of RSB occurs here even though ${q}_{max} \sim 0$. This result shall be discussed later within the context of theoretical and numerical results.

**Figure 7:**
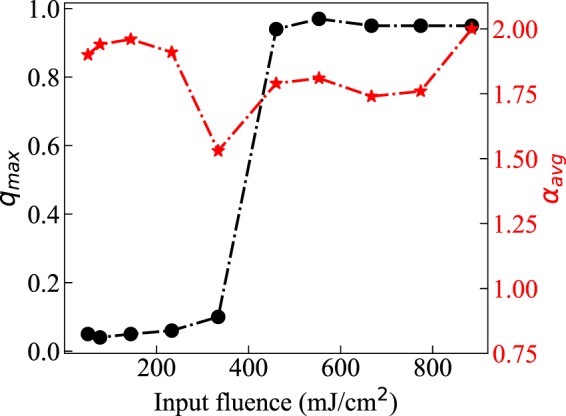
Spin-glass transition and Lévy statistics. ${q}_{max}$ representing the *q* value at which *P*(*q*) becomes maximum, along with the averaged Lévy exponent $< \alpha  >$, is plotted for different pump fluences.

Above the threshold pump intensity, *P*(*q*) distributions in Fig. 6(c,d) change their shape dramatically such that *q*_*max*_ migrates to a value close to one. This is a common signature of the spin-glass phase, where the random laser modes are presumed to oscillate in a correlated manner. The value of *q*_*max*_ is tracked with increasing pump intensity in Fig. 7 and the abrupt change of *q*_*max*_ is observed at the on-average lasing threshold, similar to prior observations^32^. In our weakly scattering ORL, however, this transition is stretched out in terms of the pump fluence so that intermittent lasing coincides with a less drastic modification to the Parisi overlap function *P*(*q*), namely its broadening. The exponent *α* described previously is now averaged and also plotted in Fig. 7. Slightly different from previous results^43^, the onset of Lévy statistics precedes that of the evidence for RSB provided by *q*_*max*_. However, if the onset of RSB actually occurs at the fluence 333.71 mJ/cm^2^ then our results are more fundamentally consistent with observation of coincidence between the two phenomena.

### Numerical results

Here, we numerically investigate random laser intensity fluctuations using similar models as Merrill *et al*.^33^, which include mode competition caused by gain saturation. Because we wish to emphasize the other requirements of RSB, like the replica condition and mode coupling, we introduce them one at a time and monitor the Parisi overlap function.

To begin, we consider completely random intensities from shot to shot where fluctuations average out. For each pump pulse, a random intensity value is assigned at wavelength index *k* and taken from the exponential statistical distribution

5 $$\rho (I(k))=\langle I\rangle (k)exp(-I(k)/\langle I\rangle (k)).$$

Since the pump pulse energy is identical for each shot, and because gain saturation is present, the total emission intensity is assumed to be constant. We determine the observed intensities for each shot by renormalizing the intensities of individual modes by the sum of the intensities across the modes as

6 $$I(k)=I(k)/\mathop{\sum }\limits_{k=1}^{N}I(k).$$

Mode competition is included via Eq. (6), however, the scattering system is effectively changing from shot to shot so that mode coupling is random and correlations between shots are absent. In other words, the replica condition is not satisfied.

**Figure 8:**
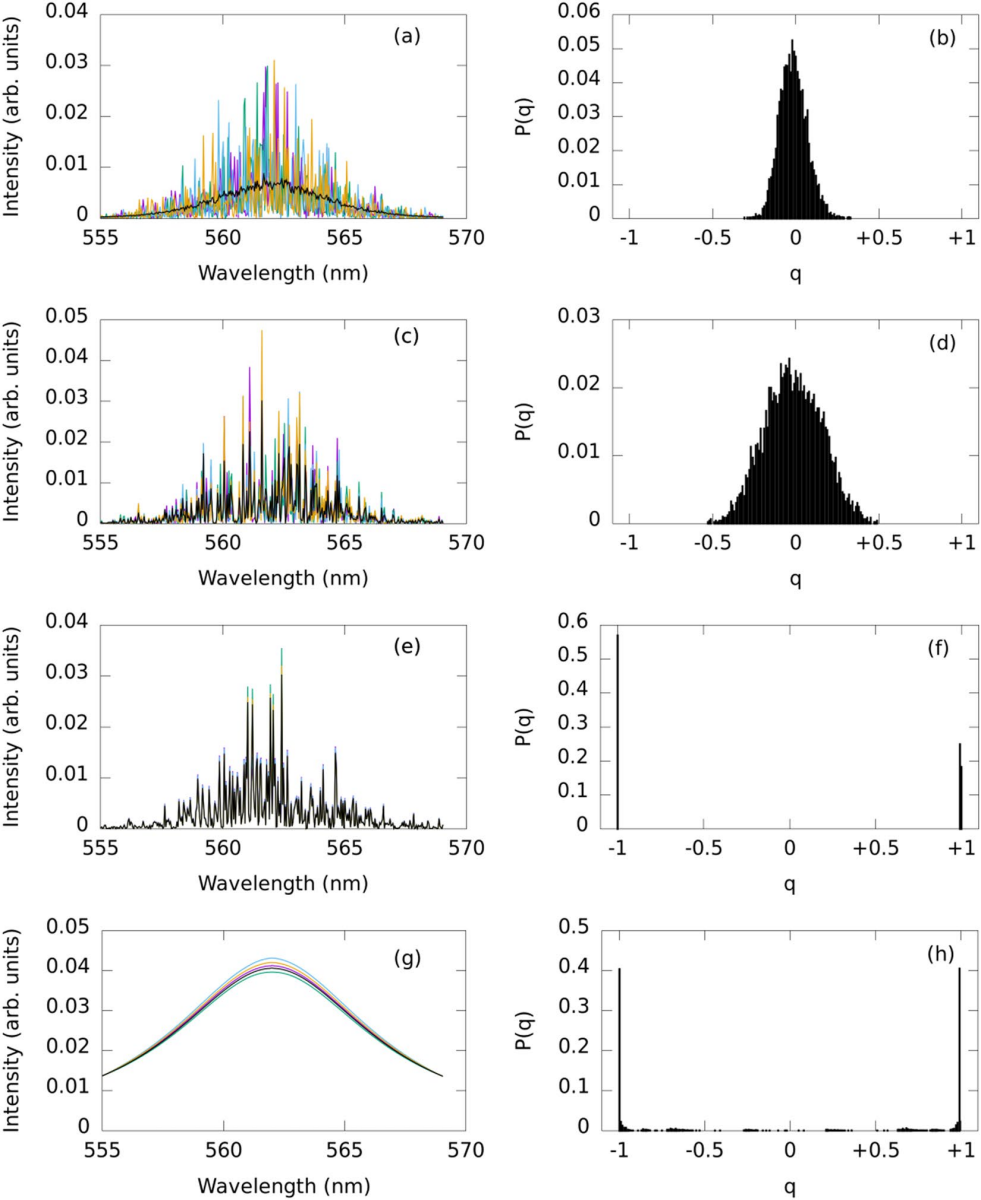
Simulation of ORL spectra with mode competition and the distribution of overlap parameter. (first column) Numerically generated emission spectra and (second column) their resulting $P(q)$ distributions for random lasers with mode competition. (**a**,**b**) Fast moving scatterers and unfrozen disorder, (**c**,**d**) frozen scatterers but lack of correlations between shots, (**e**,**f**) frozen scatterers and consistent mode coupling between shots. (**g**) Emission spectra have been simulated with random DC shifts and $B=0.1$. (**h**) The resulting distribution $P(q)$ with ${q}_{max}\ne 0$ is shown.

The intensity of a Lorentzian curve at index $k$, matching the gain curve of the ORL, is assigned to $\langle I\rangle (k)$. A few representative simulated spectra along with the total average over 100 shots is shown in Fig. 8(a). As expected, RSB is not observed in this case since ${q}_{max} \sim 0$, as shown in Fig. 8(b). Many other random spectra were calculated in a similar manner based on different random distributions, from uniform to Gaussian, with intensity normalized in each case. In all cases ${q}_{max} \sim 0$. This situation is similar to that of liquid dye-based random lasers with fast-moving mobile scatterers^33^. Lévy statistics are found in that case raising the question of what value ${q}_{max}$ would obtain. At least with this simplified numerical model, no evidence of RSB can be observed. We note that persistent mode competition is absent shot to shot, since the scattering system here changes from shot to shot. However, mode competition can still exist for the duration of the pump pulse, over which the scatterers are immobile.

Next, the *initial* intensity is chosen from the exponential distribution of Eq. (5). However, the intensity at each shot is then chosen randomly from a uniform distribution centered at the initial intensity with a $\pm 30$% range. This model now includes mode persistence due to frozen disorder, but assumes partially randomized mode coupling from shot to shot. The situation may be similar to that in the ORL experiment for the pump fluence 333.71 mJ/cm^2^, where modes persist from shot to shot only 43% of the time and different combinations of modes lase simultaneously. Figure 8(c,d) show the spectra and $P(q)$ distribution for this case. Although side lobes or shoulders are not observed, the $P(q)$ distribution does broaden and ${q}_{max}$ remains near zero.

Finally, a subset of the ${N}_{c}$ highest intensity modes in the spectra are considered positively correlated and all others negatively correlated to them (c.f., Fig. 4). Initial amplitudes are chosen from Eq. (5) and at each shot, a random amplitude fluctuation is applied with the above correlation constraints. The gain saturation condition of Eq. (6) is also maintained. Figure 8(e,f) show the spectra for ${N}_{c}=5$ and reveal evidence for RSB in this situation with ${q}_{max}\ne 0$. The result does not change qualitatively for any other values of ${N}_{c}$.

As mentioned earlier, the emission spectra in Fig. 2(e,f) exhibit DC fluctuations from shot to shot, somewhat similar to the spectral fluctuations observed in previous experiments reporting RSB in incoherent random lasers (e.g.^12,32^). Here, we simulate spectra without sharp fluctuations from wavelength to wavelength Fig. 8(g), but apply random shifts according to

7 $$I(k)=L(k)+\xi B{L}_{max}(k)\frac{|{\lambda }_{k}-{\lambda }_{0}|-\Delta {\lambda }_{max}}{\Delta {\lambda }_{max}},$$

where *L*(*k*) is a Lorentzian, $\xi$ is a random number in the range [−1, 1), and *B* is the fluctuation strength. These fluctuations effectively alter the total emission energy in each shot. However, the replica condition is only placed on the controllable experimental parameters, not the random laser emission. In this case, RSB is again indicated since $|{q}_{max}| \sim 1$, as shown in Fig. 8(h), and in agreement with previous work. We have also found (not shown) that increasing *B* broadens the peaks at $q=\pm 1$.

Additionally, we comment here that a wavelength-dependent fluctuation like Eq. (7) is not strictly necessary to observe $|{q}_{max}|\ne 0$. Maintaining the Lorentzian curve but adding purely DC fluctuations from shot to shot as $I(k)=L(k)+\xi$ was also found (not shown) to result in *q*_*max*_ values close to one instead of zero.

## Discussion

We have studied the spectral fluctuations and statistics of optofluidic random laser (ORL) emission. Randomly distributed sharp spikes appeared in the emission spectra, which cannot be predicted by the modes of a Fabry-Pérot cavity. The spectral positions of these spikes also remained fixed from shot to shot of the pump pulses. This result emphasizes the central role of random scattering and eliminates the possibility of so-called “lucky photons”^44,45^, which are seeded by spontaneous emission so that their wavelengths do not persist from shot to shot.

Typical laser threshold behavior was observed in the emission as a function of pump fluence. Correlations between ORL modes from shot to shot were observed directly by covariance measurements and can be attributed to mode coupling. Mode correlations persisted at the same set of wavelengths when the device was pumped close to and above the lasing threshold. Replica symmetry breaking (RSB) of the lasing modes was investigated using the Parisi overlap function, which peaked at non-zero values above the threshold pump power. Mode competition, demonstrated in the ORL, is a crucial factor for RSB but not the only one. Additionally, proper replicas are required, which is difficult to ensure but the experimental conditions are at least within the experimenter’s control. Other physical conditions, like the mere possibility of lasing action and mode coupling must be checked.

Lévy statistics were found to coincide with RSB, but only if *q*_*max*_ is not used as the primary indication of RSB. Prior numerical predictions and our results here support the notion that RSB may begin with more subtle, non-trivial changes to the Parisi overlap function (broadening in our case). This occurrence in the ORL is not surprising. Weak scattering means that the lasing transition is expected to be more gradual than when stronger scattering is present. Without lasing action, it is known that RSB does not occur^22^. The early occurrence of RSB at 333.71 mJ/cm^2^, before *q*_*max*_ reaches nonzero values and before the traditionally evaluated threshold ${P}_{th}=433.04$ mJ/cm^2^, is supported by the observation of Lévy statistics and the precedence that they are only observed during lasing action^41,42^. Amplified spontaneous emission, for example, does not exhibit Lévy statistics. Because a subset of the shots collected (41 of 100) at 333.71 mJ/cm^2^ does show the characteristic spikes of random lasing, we believe this fluence is close enough to threshold that lasing action occurs intermittently. Meanwhile, far above threshold, gain saturation restricts laser mode intensity and may also contribute to the lack of Lévy statistics in that region.

Numerical simulations demonstrate the strong requirement, not only for mode competition, but at least partially consistent mode coupling from shot to shot. It should be emphasized that if replica conditions are not satisfied in the experiment, unintentionally present physical mechanisms may result in spectral fluctuations that only appear as those expected from the glassy state of light.

## Methods

The ORL device was prepared by imprinting the micro-structure of positive photoresist mold AZ 9260 (Microchemicals GmbH) on a PDMS surface. First, an inverse of the final structure was fabricated on the surface of the photoresist by a photo-lithography process. This fabrication procedure has a resolution limit comparable to the wavelength of visible light, which generates an inherent randomness in the fabricated structure. In this study, we designed the final structure with a $D=3$ mm central reservoir and two $h=100$ *μ*m wide input and output channels that connected the reservoir with the inlet and outlet. To replicate the photoresist structure on the PDMS surface, PDMS and its curing agent (Sylgard 184) were mixed in a 10:1 ratio by weight. The mixture was degassed at a few mm-Hg several times after pouring on the photoresist mold. Then, the PDMS solution was cured at 90 °C for 90 min. Solid PDMS was peeled off the photoresist mold to obtain the micro-structure printed on the PDMS surface. The inlet and outlet were created in the imprinted PDMS structure to fill the dye solution. Finally, the structure was bonded on a glass slide by plasma treatment.
